# The Hippo paradox: how growth suppression drives tumor growth

**DOI:** 10.1038/s44319-026-00777-6

**Published:** 2026-05-01

**Authors:** Pooja Rai, Andreas Bergmann

**Affiliations:** UMass Chan Medical School, Department of Molecular, Cell and Cancer Biology, Worcester, MA USA

**Keywords:** Cancer, Signal Transduction

## Abstract

A study in this issue challenges the prevailing unidirectional view of Hippo signaling as a purely tumor-suppressive pathway by showing that the outcome of Hippo activation is highly context-dependent in the Drosophila wing disc.

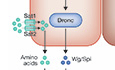

## Teaser

The Hippo pathway is classically viewed as a tumor suppressor that limits tissue growth by inhibiting the transcriptional co-activator Yorkie/YAP. Accordingly, Hippo activation is expected to suppress proliferation and can promote apoptosis. In this issue, Honda and colleagues challenge the prevailing unidirectional view of Hippo signaling as a purely tumor-suppressive pathway by showing that the outcome of Hippo activation is highly context-dependent in the *Drosophila* wing disc (Honda et al, 2026). In the wing pouch, Hippo activation leads primarily to growth suppression, as expected. In contrast, in the hinge and ventral notum, Hippo-activated cells survive and act as “oncogenic niche” cells that stimulate tumorigenic proliferation in neighboring cells. This tumor-promoting effect is mediated by non-apoptotic activity of the initiator caspase Dronc, induction of Wnt and EGF signaling, and metabolic support provided by the amino-acid transporters Sat1 and Sat2, which together drive mTOR activation in nearby cells. These findings reveal how growth suppression in one cell population can paradoxically drive tumor growth in another, reframing Hippo activity as a regulator of tumor-promoting microenvironments.

**Figure 1:**
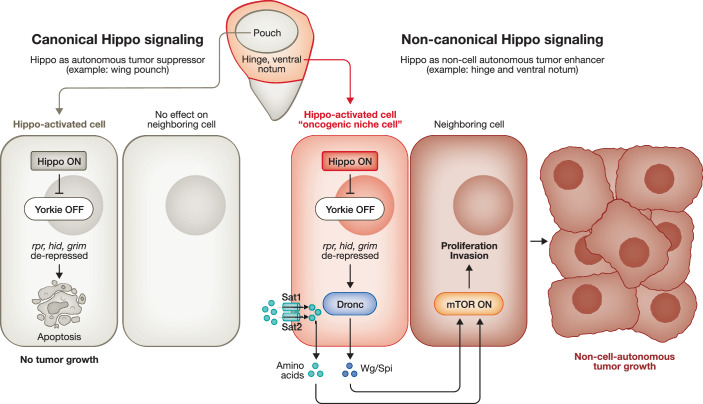
Canonical and noncanonical Hippo signaling in the larval wing imaginal disc. Left: canonical Hippo signaling. When the Hippo pathway is activated in the pouch region of the wing disc, Yorkie (Yki) is inhibited, resulting in derepression of the pro-apoptotic genes *reaper*, *hid*, and *grim* and induction of apoptosis. In this cell-autonomous context, Hippo activation suppresses tissue growth and tumor formation, and no proliferative signals are transmitted to neighboring cells. Right: noncanonical Hippo signaling. Hippo-activated cells located in the hinge/ventral notum region of the wing disc behave as “oncogenic niche cells.” These cells redirect the activity of the initiator caspase Dronc to adopt a non-apoptotic signaling function. In this role, Dronc induces secretion of the mitogens Wingless (Wg) and Spitz (Spi), while the amino acid transporters Sat1 and Sat2 promote metabolic support. These signals activate mTOR signaling in neighboring cells, resulting in non-cell-autonomous proliferation and tumor growth. This model illustrates how a tumor suppressor pathway can paradoxically promote tumor growth through non-cell-autonomous signaling and metabolic coupling.

The Hippo pathway is an evolutionarily conserved tumor suppressor pathway that was discovered in *Drosophila* and is also present in mammals. It controls tissue growth and organ size through the transcriptional co-activators Yorkie (Yki) in flies and its mammalian homolog YAP (Zheng and Pan, 2019). When Hippo signaling is inactive, Yki/YAP translocates into the nucleus and induces expression of genes that promote proliferation and cell survival, resulting in tissue overgrowth and tumorigenesis. This form of tumor growth is strictly cell-autonomous. Only cells with active Yki/YAP proliferate and contribute to tumor formation. In contrast, activation of the Hippo kinase cascade (generating “Hippo-activated cells”) results in phosphorylation and cytoplasmic retention of Yki/YAP, thereby preventing the expression of genes that promote proliferation, and de-repressing genes that can induce apoptosis, such as the pro-apoptotic genes *reaper* (*rpr*), *hid*, and *grim* (Fig. 1). As a result, Hippo-activated cells typically undergo growth arrest or apoptosis (Zheng and Pan, 2019). Thus, in this canonical view, loss of Hippo signaling promotes tumor growth, whereas Hippo activation suppresses growth and can eliminate cells. Hence, Hippo-activated cells are not expected to contribute to tumor formation.

The study by Honda and colleagues reveals a strikingly different scenario (Honda et al, 2026). These authors observed that tissues containing Hippo-activated cells can nevertheless develop tumor-like overgrowth, contrary to expectations. Importantly, a closer inspection revealed that the outcome of Hippo activation is highly context-dependent, with the same genetic manipulation resulting in either growth suppression or tumor formation depending on its location within the *Drosophila* wing disc. When Hippo-activated cells are generated in the wing pouch, the expected tumor-suppressive outcome is observed (Fig. 1). In contrast, when Hippo-activated cells are located in the hinge and ventral notum regions of the wing disc, tissues instead develop tumor-like masses (Honda et al, 2026). Notably, the Hippo-activated cells themselves do not overproliferate. Rather, tumor growth occurs in neighboring or nearby cells (Fig. 1), indicating that Hippo-activated cells can promote tumor formation in a non-cell-autonomous manner (Honda et al, 2026). Thus, depending on tissue context, Hippo activation can lead either to growth suppression or, paradoxically, to tumor growth in surrounding tissue.

How can this paradoxical behavior be explained? Importantly, the initial molecular events in Hippo-activated cells appear to be similar regardless of whether the outcome is growth suppression or tumor formation. In both cases, activation of the Hippo pathway inhibits Yki activity, resulting in the derepression of the pro-apoptotic genes *rpr*, *hid*, and *grim* and subsequent activation of caspases, most notably the initiator caspase Dronc (Fig. 1). Under normal conditions, Dronc activation triggers apoptosis, as observed in the wing pouch, where Hippo activation results in growth suppression. However, in the hinge and ventral notum, Dronc activity does not lead to widespread apoptosis. Instead, Dronc activity is redirected toward a non-apoptotic signaling function, and Hippo-activated cells persist and become an “oncogenic niche”. In this role, they generate mitogenic signals such as Wingless (Wg, a Wnt ortholog) and Spitz (Spi, an EGF ortholog), which activate proliferative, invasive, and mTOR-dependent signaling in adjacent cells (Fig. 1) (Honda et al, 2026). Thus, while the upstream Hippo pathway activity is similar in different regions of the tissue, the downstream outputs of caspase activation diverge, leading either to cell death or to the formation of a tumor-promoting niche.

The behavior of Hippo-activated cells bears strong resemblance to apoptosis-induced proliferation (AiP), in which non-apoptotic activity of Dronc in dying or “undead” cells stimulates proliferation in neighboring tissue through the secretion of the same mitogens Wg and Spi (Bergmann, 2025; Bergmann and Fan, 2025). However, Dronc-Wg/Spi signaling alone cannot fully explain the robust non-cell-autonomous mTOR activation observed (Honda et al, 2026), suggesting that additional Hippo-dependent mechanisms contribute to tumor growth.

Key additional components identified in this study are the amino acid transporters Sat1 and Sat2, homologs of mammalian SLC36 transporters (Honda et al, 2026). These transporters regulate amino acid availability and are required for non-cell-autonomous mTOR activation (Fig. 1). These data suggest a model in which Hippo-activated cells increase amino acid availability in neighboring cells. Amino acids may originate from increased uptake by Sat1 and Sat2 into Hippo-activated cells, and are then transported into neighboring cells where they activate mTOR signaling and drive proliferation (Fig. 1) (Honda et al, 2026). In this way, Hippo-activated cells not only provide mitogenic signals but also metabolic support, which creates a tumor-supporting microenvironment.

Collectively, this work positions Hippo-activated oncogenic niche signaling within the broader context of non-apoptotic caspase functions. It establishes a unified mechanism of non-cell-autonomous tumorigenesis that integrates caspase signaling, growth factor output, and metabolic coupling, thereby extending well beyond the primary reliance on mitogen secretion of classical AiP.

This study raises several important questions. One key question is what determines the striking regional specificity of Hippo activation, such that Hippo-activated cells act as tumor suppressors in the wing pouch but as oncogenic niche cells in the hinge and ventral notum. In both regions, Hippo activation leads to activation of Dronc, yet the cellular outcomes differ dramatically, resulting in apoptosis in the pouch but survival and signaling activity in the hinge and ventral notum (Honda et al, 2026). This suggests that the critical difference lies not in Hippo pathway activation itself, but in how Dronc activity is interpreted by the cell. One possible explanation is that Dronc may be spatially restricted or relocalized within the cell, for example, to the plasma membrane, as observed in AiP (Amcheslavsky et al, 2018; Braun et al, 2025), where membrane-associated Dronc promotes mitogen production without triggering apoptosis. However, this raises a further question: why would such a redirection of Dronc activity occur in the hinge and ventral notum, allowing Hippo-activated cells to survive and signal, but not in the pouch? Identifying the tissue-specific factors that determine whether caspase activity leads to cell death or to intercellular signaling will be an important goal for future studies.

The implications of this work extend beyond *Drosophila*. In several human cancers, YAP activity is reduced rather than increased, and components of the Hippo pathway can display tumor-promoting functions in vivo (Cottini et al, 2014; Moroishi et al, 2016; Pearson et al, 2021; Yuan et al, 2008). This study provides a mechanistic explanation for this paradox by showing that Hippo-activated cells can promote tumor growth indirectly by acting on the tumor microenvironment (Honda et al, 2026). Thus, the Hippo pathway can function both as a tumor suppressor and as a tumor promoter, depending on whether its effects are cell-autonomous or mediated through neighboring cells.

Together, this work reveals a surprising principle: a growth-suppressive pathway can paradoxically promote tumor growth by creating an oncogenic niche that stimulates proliferation and metabolic activation in surrounding cells. These findings highlight the importance of non-cell-autonomous signaling and metabolic coupling in tumorigenesis and suggest that targeting tumor-microenvironment interactions may be as important as targeting tumor cells themselves.
